# Screening for candidate genes involved in the production of mouse subventricular zone proliferative cells and an estimation of their changes in evolutionary pressure during primate evolution

**DOI:** 10.3389/fnana.2013.00024

**Published:** 2013-07-31

**Authors:** Hidenori Tabata, Tsuyoshi Hachiya, Koh-ichi Nagata, Yasubumi Sakakibara, Kazunori Nakajima

**Affiliations:** ^1^Department of Anatomy, School of Medicine, Keio UniversityTokyo, Japan; ^2^Department of Molecular Neurobiology, Institute for Developmental Research, Aichi Human Service CenterKasugai, Japan; ^3^Department of Biosciences and Informatics, Keio UniversityYokohama, Japan; ^4^Iwate Medical Megabank Organization, Iwate Medical UniversityShiwa, Japan

**Keywords:** neural stem cells, human evolution, notch signaling, selection bias, subventricular zone (SVZ)

## Abstract

During neocortical development, excitatory neurons are produced from apical progenitors in the ventricular zone (VZ) or from dividing cells in the subventricular zone (SVZ). We previously reported that the direct progenies of VZ cells in mice slowly exit the VZ and accumulate just above the VZ (lower SVZ) as multipolar migrating neurons, whereas subsequently dividing cells in the SVZ exit the VZ earlier than the former and become widely distributed in the SVZ. These two populations are named the slowly exiting population (SEP) and the rapidly exiting population (REP), respectively. In mice, REP cells include basal progenitors as the major population and are characterized by a long ascending process; their morphology resembles that of basal radial glial cells (bRGs), which have been observed in the inner and outer SVZ in primates. The dramatic increase in the number of bRGs in primates, especially in humans, is thought to underlie the acquisition of a huge cortex during evolution. We previously reported that the REP/SEP production rate in the lateral cortical VZ is higher than that in the dorsomedial VZ in mice. To search for molecules responsible for the higher REP production in the lateral cortical VZ, we conducted microarray analyses and identified genes that were differentially expressed between the lateral and medial VZs in mice. These genes were considered to be among the candidates responsible for the regulation of the REP/SEP production rate. To investigate the selection pressures during primate evolution on these candidate genes, we estimated the synonymous vs. non-synonymous base substitution rates. As a result, the negative selection pressures on the *Megf11, Dmrt3*, and *Cntn3* genes were found to be significantly weaker in primates than in non-primates, while those on *Jag1, Ntrk2*, and *Pmp22* were stronger. Candidate molecules responsible for primate cortical expansion through an increase in bRGs may be included among these genes.

## Introduction

During evolution, humans have acquired a huge cerebral cortex that is thought to be the base of our higher mental activities. The cortical neurons in mammals are generated from the ventricular zone (VZ) lining the lateral ventricles or the adjacent subventricular zone (SVZ) during the embryonic or fetal stages. In murine embryos, neural stem cells exist in the VZ and are called radial glia (RG) (Noctor et al., 2001). These cells extend a long, ascending (basal) process, which is also called a radial fiber, toward the pial surface while a short thick apical process reaches the ventricular surface. During the neurogenic cell divisions, RG undergo asymmetric cell divisions to produce one RG and one neuron. In many instances of this asymmetric cell division, the daughter cells that have inherited the ascending process become RG, especially during mid or late cortical development (Konno et al., 2007). RG also produce proliferative cells in the SVZ, and most of the proliferative cells in the murine SVZ undergo symmetric cell division to produce two neurons (Noctor et al., 2004). This cell division is terminal, and this type of progenitor cells in the SVZ is called the basal progenitor (BP). They are proliferative but do not have self-renewing ability and contribute to temporal amplification of neuron production. In that sense, they are also called intermediate progenitor cells (IPCs).

We previously observed the initial phase of the migration of cells exiting the VZ during late cortical plate development in mice (embryonic day [E] 14.5~16) and described two distinct populations (Tabata et al., 2009). One population finishes their final cell division in the VZ, remains there for more than 10 h, and then exits from the VZ and accumulates in the lower SVZ, where the cells exhibit a multipolar cell morphology (Tabata and Nakajima, 2003). We named this region the multipolar cell accumulation zone (MAZ) to avoid confusion with the term SVZ, which is defined as the region containing proliferative cells not attached to the ventricular surface and not showing interkinetic nuclear movement during the mitotic cycle (Boulder Committee, 1970). After remaining in the MAZ for about 24 h, the multipolar neurons then extend an axon tangentially and resume their migration using a locomotion mode in which the cells assume a bipolar morphology with a thick leading process and a thin trailing process and migrate using the radial fiber as a scaffold. The other population exits from the VZ faster than the former population and uses a somal translocation mode in which cells with a long ascending process retract this process to translocate the cell body while the tip of the ascending process remains attached to the brain surface; these cells divide in the intermediate zone (IZ) or SVZ. As these cell populations differ from each other in the timing of their exit from the VZ, we named them the slowly exiting population (SEP) and the rapidly exiting population (REP), respectively. Because the SEP includes all the cells that have completed cell division while in the VZ, it includes the daughter cells of short neural progenitors (SNPs), which have a short ascending fiber in the VZ and produce two neurons as the apical intermediate progenitors (Gal et al., 2006). Among the REP cells, 7.6% expressed Olig2, a glial progenitor marker, and 65% expressed NeuroD, a neuronal marker, at 36 h after the introduction of a GFP expression vector using *in utero* electroporation (Tabata et al., 2009). These results indicated that the REP includes some glial progenitors, while the major population is neurogenic.

In primate fetal cortices, including those of humans, the SVZ becomes enormously thick as the development proceeds and is divided into two histologically distinct regions, the outer SVZ (OSVZ) and the inner SVZ (ISVZ) (Smart et al., 2002). The OSVZ is located superficially to the ISVZ and shows a columnar organization of cells, whereas the ISVZ consists of randomly oriented cells. Interestingly, the thickness of the OSVZ increases during development, while that of the ISVZ is rather constant, indicating that the OSVZ is the major source of the cortical neurons, at least during late cortical plate (CP) development. Recently, Hansen et al. and Fietz et al. demonstrated the existence of a novel type of progenitor cell in the human OSVZ (Fietz et al., 2010; Hansen et al., 2010). This type of progenitor cells has a long ascending process and expresses stem cell markers such as Pax6, Hes1, and Sox2. During the mitosis of this cell type, the ascending process is maintained and is inherited by one of the daughter cells, which is then able to further divide in the same manner. Thus, this type of cells undergoes self-renewing asymmetric cell divisions. These morphological and behavioral features strikingly resemble those of RG in the VZ. Accordingly, Hansen et al. referred to these cells as OSVZ radial glia-like (oRG) cells. They originally postulated that oRG cells might have newly appeared during primate evolution. However, oRG cells were later also found in other mammalian species, including ferrets, rats and mice (Fietz et al., 2010; Reillo et al., 2011; Shitamukai et al., 2011; Wang et al., 2011; Kelava et al., 2012; Martínez-Cerdeño et al., 2012), and they were found not only in the OSVZ, but also in the ISVZ in ferrets and marmosets (Reillo et al., 2011; García-Moreno et al., 2012). Therefore, this cell type is also called basal RG (bRG) (Englund et al., 2005). It is generally thought that the dramatic increase in the number of bRGs in the primate fetal cortex, especially in humans, may underlie the acquisition of a large cerebral cortex during evolution.

After the description of human OSVZ cells by Hansen et al., we noticed that the histological features of the mouse IZ/SVZ were well-correlated with the primate SVZ as follows (Tabata et al., 2012). First, the mouse MAZ and the primate ISVZ are both located just above the VZ and are composed of highly packed and randomly oriented cells. Second, both the mouse REP cells and the primate bRG cells have a long ascending process, exhibit proliferative activity, and are located widely in the SVZ/IZ from just above the VZ to the IZ. The mouse REP is not equal to mouse bRGs. Rather, REP includes BPs as the major population and lessor Olig2-positive glial progenitors in addition to the self-renewed mouse bRGs. All these cell types in the REP have a long ascending process, at least before the first cell division in the SVZ. The cells with this morphology, including Olig2-positive glial progenitors, are called translocating RG (tRG) (Martínez-Cerdeño et al., 2012). We observed that BPs among the REP also have the tRG morphology before cell division in the SVZ/IZ and that they were indistinguishable from bRG morphologically, suggesting that BPs and bRG might not be completely distinct cell types, but instead might only differ in the degree of their undifferentiated states. Hence, we hypothesized that the expansion of the SVZ during brain evolution in primates might have been caused by an increase in REP production and/or maintenance rates in primates, compared with that in rodents. Therefore, we assumed that the molecular mechanisms responsible for the regulation of the REP/SEP production ratio might have evolved and been promoted in primates in a manner such that the number of bRGs was increased.

In mice, we previously observed that the REP production rate, compared with the SEP production rate, was relatively high in the lateral cortex and low in the dorsomedial cortex (Tabata et al., 2009). This fact is probably due to the need of the lateral VZ to produce more neurons destined to reach the laterally extended CP outside the lateral edge of the cortical VZ. Whatever the reason for this difference in production rates in mice, we expected that genes regulating the higher REP/SEP production ratio in the mouse lateral VZ might be among the candidates genes responsible for the increased generation of bRGs in primate cortex, leading to the acquisition of a huge cerebral cortex, during evolution.

Here, we compared the expression profiles between lateral and dorsomedial VZ cells in mice during late cortical plate development. The gene expression profiles were analyzed using Affymetrix GeneChip microarrays. This screening of differentially expressed genes combined with validation using *in*-*situ*-hybridization experiments identified 29 and 9 genes with higher expression levels in the lateral and dorsomedial cortices, respectively. We then performed detailed bioinformatics evolutionary analyses. As a result, significant changes were found for six genes: the negative selection pressures on *Megf11, Dmrt3*, and *Cntn3* were weaker in primate lineages than in non-primate lineages, whereas those on *Jag1, Ntrk2*, and *Pmp22* were stronger in primate lineages than in non-primate lineages. Candidate molecules responsible for primate cortical expansion through increase of bRGs may be included in these genes.

## Materials and methods

### Animals

Pregnant ICR mice were purchased from Japan SLC. The day of vaginal plug detection was considered embryonic day 0 (E0). All the animal experiments were performed according to the Guidelines for the Care and Use of Laboratory Animals of Keio University School of Medicine.

### Fluorescence-activating cell sorting (FACS) of the lateral and dorsomedial VZ cells

To label the differentiation states of the lateral and dorsomedial VZ cells, we introduced a green fluorescent protein (GFP) expression vector at E15.5 using *in utero* electroporation. The vector we used expresses enhanced GFP (EGFP) cDNA under the control of the CAG promoter, which contains the cytomegalovirus early enhancer and chicken beta-actin promoter (a kind gift from Dr. Jun-ichi Miyazaki, Osaka University) (Niwa et al., 1991). *In utero* electroporation was performed as described previously (Tabata and Nakajima, 2001). Briefly, we injected 3 μg/μ L of plasmid solution into the lateral ventricles and applied 35-V, 50-ms pulses 4 times using an electroporator (CUY-21E or NEPA21; NEPA GENE) with a forceps-type electrode (CUY650P5). To label the lateral and dorosomedial VZ cells differentially, we changed the direction of the electrode.

At 18 h after the electroporation, the brains were removed and the GFP-positive area was dissected out under a dissection microscope with a fluorescent lamp. The tissue fragments were treated with trypsin and DNaseI and then triturated by pipetting in DMEM with 10% FCS. GFP-positive cells were isolated using EPICS ALTRA HyPerSort and Expo 32 software (Beckman Coulter). Dissociated cells were stained with propidium iodide (PI) prior to sorting. The gating parameters were determined using the side and forward scatter to eliminate debris and aggregated cells, using red fluorescence (610 nm) to eliminate dead cells, and using green fluorescence (488 nm) to separate the positive from negative cells (cells prepared from un-injected brains were used as the negative control).

The sorted cells were directly collected in Trizol (Invitrogen). We performed FACS 3 times independently and pooled all the samples, collecting about 5 × 10^5^ cells for each sample in total. The total RNA was extracted from each sample. The quality control and an estimation of the extracted total RNA concentration were performed using an Agilent 2100 Bioanalyzer (Agilent Technologies).

### Genechip expression analysis

About 130 ng of total RNA was used to prepare biotinylated cRNAs, which were hybridized to Affymetrix GeneChip Mouse Expression Set 430 2.0 arrays. The GeneChip data analysis was performed as described previously (Tachikawa et al., 2008). Briefly, the hybridized microarray images were analyzed using the Affymetrix Microarray Software 5.0 (MAS5.0) algorithm. The detection calls, change calls, and the signal log ratios were determined using the Expression Console Version 1.1 (Affymetrix). We classified the candidate genes using gene ontologies (GOs) and the Protein Analysis Through Evolutionary Relationships (PANTHER) database analysis software (SRI International, Menlo Park, CA, http://www.pantherdb.org).

### *In situ* hybridization

E16 ICR mouse embryos were fixed using perfusion with 4% paraformaldehyde (PFA) in 0.1 M sodium phosphate buffer (pH7.4). The brains were removed and post-fixed in the same fixative overnight at 4°C. The brains were then equilibrated in 30% sucrose in PBS and embedded in OCT compound (Sakura Finetek). Frozen sections were cut coronally in 20-μ m thick sections using a cryostat (CM1900; Leica) and were mounted onto MAS-coated glass slides (Matsunami). To prepare the RNA probes, the plasmid vectors of the RIKEN FANTOM clone set were digested with appropriate restriction enzymes or PCR products amplified from the RIKEN FANTOM clone set using a primer pair (forward primer M13-21, 5′-TGTAAAACGACGGCCAGT-3′; reverse primer 1233, 5′-AGCGGATAACAATTTCACACAGGA-3′) as the template for *in vitro* transcription. The clones that were used are listed in Table 1. For *Dmrt3*, we used GeneCopoeia GC-Mm23549 as the template DNA. Digoxigenin (DIG)-labeled RNA probes were synthesized using T7 or T3 RNA polymerase and the DIG RNA labeling kit (Roche). Sections were treated with 4% PFA and acetylated with 100-mM triethanolamine (pH8.0). Pre-hybridization was performed in 50% formamide, 5x SSPE, 5% SDS, and 1 mg/mL of yeast transfer RNA (Sigma) at 55°C for 1 h. Hybridization was performed in the same solution with 1 μ g/mL of a DIG-labeled RNA probe at 55°C overnight. The hybridized probes were reacted with alkaline phosphatase-conjugated anti-DIG antibody (1:2000; Roche). Signals were developed in alkaline phosphatase buffer containing nitroblue tetrazolium chloride (NBT) and 5-bromo-4-chloro-3-indolyl-phosphate (BCIP).

**Table 1:** **29 lateral-high and 9 medial-high candidate genes**.

**Probe set ID**	**Clone ID**	**Entrez Gene ID**	**Signal (lateral)**	**Signal (medial)**	**Signal Log Ratio**	**Gene title**	**Gene symbol**	**Gene ontology (Biological Process)**	**Gene ontology (Cellular Component)**
1449286_at	GeneSat only	80883	585.9	57.1	3.2	Netrin G1	Ntng1	Nervous system development//cell differentiation	plasma membrane
1450992_a_at	PX01269N19	17268	887.7	155.9	2.3	Meis homeobox 1	Meis1	Regulation of transcription	nucleus
1420838_at	FW00008O14	18212	4035.7	954.2	2.3	Neurotrophic tyrosine kinase, receptor, type 2	Ntrk2	Transmembrane receptor protein tyrosine kinase signaling pathway//nervous system development	cytosol
1447640_s_at	PL00253I02	18516	2924.0	643.6	2.2	Pre B-cell leukemia transcription factor 3	Pbx3	Regulation of transcription//neuron development	nucleus
1417420_at	PS00062L11	12443	1210.0	280.0	2.1	Cyclin D1	Ccnd1	Cell cycle//negative regulation of Wnt receptor signaling pathway	nucleus//cytosol
1434070_at	PX01236J19	16449	1483.8	362.3	1.9	Jagged 1	Jag1	Notch signaling pathway//negative regulation of cell differentiation	plasma membrane
1435605_at	PX00950H24	242894	1868.4	513.0	1.8	ARP3 actin-related protein 3 homolog B (yeast)	Actr3b	Regulation of actin filament polymerization	cytoplasm
1417520_at	PX01253O18	18025	391.9	145.4	1.8	Nuclear factor, erythroid derived 2, like 3	Nfe2l3	Regulation of transcription	nucleus
1417133_at	GeneSat only	18858	2657.2	803.8	1.7	Peripheral myelin protein 22	Pmp22	Cell cycle//myelin formation	membrane
1456395_at	PS00059L19	19017	205.2	94.1	1.7	Peroxisome proliferative activated receptor, gamma, coactivator 1 alpha	Ppargc1a	Positive regulation of transcription	nucleus
1441059_at	PX01259J17	73430	81.7	28.8	1.6	RIKEN cDNA 1700049G17 gene (1700049G17Rik), mRNA	1700049G17Rik		intracellular
1438628_x_at	PX01269N15	18488	247.1	87.5	1.6	Contactin 3	Cntn3	Cell adhesion	plasma membrane
1450165_at	PX01237J21	20556	177.2	50.5	1.5	Schlafen 2	Slfn2	Negative regulation of cell proliferation	
1421943_at	PX01260A16	21802	501.0	165.4	1.5	Transforming growth factor alpha	Tgfa	Activation of MAPK activity//cell proliferation//positive regulation of epidermal growth factor receptor activity	extracellular region
1433842_at	PX00963H03	16978	806.4	324.2	1.4	Leucine rich repeat (in FLII) interacting protein 1	Lrrfip1	Regulation of transcription	nucleus
1437422_at	PX01272I12	20356	2756.4	1092.2	1.4	Sema domain, seven thrombospondin repeats (type 1 and type 1-like), transmembrane domain (TM) and short cytoplasmic domain, (semaphorin) 5A	Sema5a	Patterning of blood vessels//nervous system development//axon guidance	membrane
1453055_at	PX00986D16	214968	2032.3	639.1	1.4	Sema domain, transmembrane domain (TM), and cytoplasmic domain, (semaphorin) 6D	Sema6d	Nervous system development//cell differentiation	membrane
1433489_s_at	GeneSat only	14183	2214.8	901.3	1.3	Fibroblast growth factor receptor 2	Fgfr2	Positive regulation of cell proliferation//fibroblast growth factor receptor signaling pathway	membrane
1419402_at	PX01250J14	17427	151.4	56.4	1.3	meiosis-specific nuclear structural protein 1	Mns1	Meiosis	nucleus
1418157_at	PX01256A06	100046044 /// 13865	8497.2	2995.6	1.3	Similar to COUP-TFI///nuclear receptor subfamily 2, group F, member 1	LOC100046044 /// Nr2f1	Negative regulation of transcription//neuron migration	nucleus
1434430_s_at	PX00973H03	100045233 /// 11541	94.7	34.1	1.2	adenosine A2b receptor/// similar to Adenosine A2b receptor	Adora2b /// LOC100045233	G-protein coupled receptor protein signaling pathway// positive regulation of cAMP biosynthetic process	plasma membrane
1450017_at	PL00261I13	12450	320.9	141.6	1.2	Cyclin G1	Ccng1	Cell cycle	nucleus
1453119_at	PX00624I04	71198	317.3	118.1	1.2	OTU domain containing 1	Otud1		
1440423_at	PX00988I10	240869	116.8	51.0	1.2	Zinc finger and BTB domain containing 37	Zbtb37		intracellular
1458015_at	PX00984I24	214058	333.3	120.4	1.1	Multiple EGF-like-domains 11	Megf11		plasma membrane
1443378_s_at	PX01228H21	280668	351.8	159.5	1.0	A disintegrin and metallopeptidase domain 1a	Adam1a	Proteolysis//fusion of sperm to egg plasma membrane	membrane
1439205_at	PX01006E22	18019	251.1	131.0	1.0	Nuclear factor of activated T-cells, cytoplasmic, calcineurin-dependent 2	Nfatc2	Cytokine production//positive regulation of transcription	nucleus
1430583_at	PX01007O14	319504	2604.7	1273.1	1.0	Neuron-glia-CAM-related cell adhesion molecule	Nrcam	Cell adhesion//central nervous system development//cell-cell adhesion	plasma membrane
1450905_at	PX00987H09	54712	650.4	288.0	1.0	Plexin C1	Plxnc1	Signal transduction	membrane
1417667_a_at	ZX00138B04	19212	597.9	1386.9	−1.1	Phosphotriesterase related	Pter	catabolic process	
1418106_at	PS00060N16	15214	62.0	157.0	−1.1	Hairy/enhancer-of-split related with YRPW motif 2	Hey2	Regulation of transcription//negative regulation of Notch signaling pathway	nucleus
1449581_at	PX01264M24	140703	807.2	1859.4	−1.2	EMI domain containing 1	Emid1		extracellular region
1436694_s_at	GeneSat only	11923	391.2	1004.7	−1.2	Neurogenic differentiation 4	Neurod4	Regulation of transcription//Notch signaling pathway//nervous system development	nucleus
1427529_at	GeneSat only	14371	165.4	351.8	−1.2	Frizzled homolog 9 (Drosophila)	Fzd9	G-protein coupled receptor protein signaling pathway//neuroblast proliferation//Wnt receptor signaling pathway	plasma membrane
1428662_a_at	ZA00031E02	74318	1138.0	2919.4	−1.4	HOP homeobox	Hopx	Negative regulation of transcription//histone deacetylation//negative regulation of cell differentiation	nucleus
1460412_at	GeneSat only	70370	114.6	280.8	−1.4	Fibulin 7	Fbln7	Cell adhesion	extracellular region
1440707_at	GeneCopoeia GC-Mm23549	240590	195.0	858.7	−1.8	Doublesex and mab-3 related transcription factor 3	Dmrt3	Regulation of transcription//sex determination//cell differentiation	nucleus
1416630_at	ZA00025J24	15903	182.9	1803.9	−3.0	Inhibitor of DNA binding 3	Id3	Negative regulation of transcription//neuron differentiation	nucleus

Probe set ID, Affymetrix Mouse Expression Set 430 Probe Set ID. If multiple probe sets correspond to one gene, the one that of signal value is highest was listed. Clone ID, The FANTOM clone rearray ID used to prepare RNA probe for in situ ohybridization, “GeneSat only” indicates the genes that of expression pattern was confirmed only in GeneSat databese. As to Dmrt3, the probe for in situ hybridization was prepared from the indicated clone of GeneCopoeia; Signal (lateral and medial), The normalized signal intensity of each gene after the hybridization of transcripts from lateral and dorsomedial VZ cells; Signal Log Ratio, The difference of signal intensity represented by log_2_(lateral/medial); Gene Ontology (Biological Process) and (Cellular Component), Major functions and location of each protein described in GeneOntology data.

### Bioinformatics evolutionary analyses

We performed bioinformatics evolutionary analyses for 38 candidate genes that are differentially expressed between lateral and dorsomedial VZ cells in mice. To determine the genes of other species that are orthologous to these 38 candidate genes in mice, one-to-one orthologous relationships were obtained from the EnsemblCompara DB (Vilella et al., 2009). We accepted only one-to-one orthologous relationships so as to avoid the inclusion of paralogous genes in our evolutionary analyses. We compared the coding sequences (CDS) of genes in 13 mammals (human, chimpanzee, orangutan, macaque, mouse, rat, rabbit, dog, cow, armadillo, elephant, tenrec, and opossum) as well as those of the anole lizard as an out-group. Although genome information for other vertebrates is available, we did not include such species because of poor-quality genome assembly and/or gene annotations. Reliable genomic information was selected according to the “13 eutherian mammals EPO” dataset in Ensembl (http://asia.ensembl.org/info/docs/compara/analyses.html).

Given the CDS of orthologous genes in the 14 species, multiple alignments were calculated using PRANK+F (Löytynoja and Goldman, 2008) based on the phylogenetic tree shown in Figure 3A. Alignment columns containing gaps were removed, as described previously (Tárraga et al., 2007; Capella-Gutiérrez et al., 2009).

To elucidate the evolutionary pressure on the candidate genes, we estimated the dN/dS ratios based on evolutionary models. The first model contained two ω (= dN/dS) parameters (Yang, 1998): one ω was for primate lineages, and the other was for non-primate lineages. This means that the dN/dS ratio was constant among primate and non-primate lineages, respectively. Because the model uses two ω (= dN/dS) parameters, the dN/dS ratios can differ between primate and non-primate lineages. The model also assumed that the ω ratio was constant across whole amino acid residues of a gene product. We denoted this evolutionary model as the “two-ratio” model.

To test whether the dN/dS ratio of primate lineages differs significantly from that of non-primate lineages, an alternative evolutionary model with one ω parameter was also used (Yang, 1998). This model assumed that the dN/dS ratio was constant for all lineages of the phylogenetic tree shown in Figure 3A and that the dN/dS ratio was constant across whole amino acid residues of a gene product. This model was called the “one-ratio” model. To determine which of the one-ratio or the two-ratio models had a better fit with a given alignment, a likelihood ratio test (Yang, 1998) was applied. If the two-ratio model was accepted, this finding would suggest that the dN/dS ratio of primate lineages differed significantly from that of non-primate lineages. The calculation of the likelihood ratio test was conducted using PAML4 (Yang, 2007).

For detailed evolutionary analyses, we employed *branch-site models*, which relax the assumption of the above one-ratio and two-ratio models and allow the ω ratio to vary both among lineages and among amino acid sites (Yang and Nielsen, 2002; Yang et al., 2005; Zhang et al., 2005). The branch-site test of positive selection was applied to given alignments as described in PAML (Yang, 2007). Amino acid residues under positive selection were predicted using the Bayes empirical Bayes (BEB) method (Yang et al., 2005). The calculation of the branch-site test of positive selection was executed by PAML4 (Yang, 2007).

## Results

### Screening of laterally and medially expressed genes in mice during late cortical plate development

**Figure 1:**
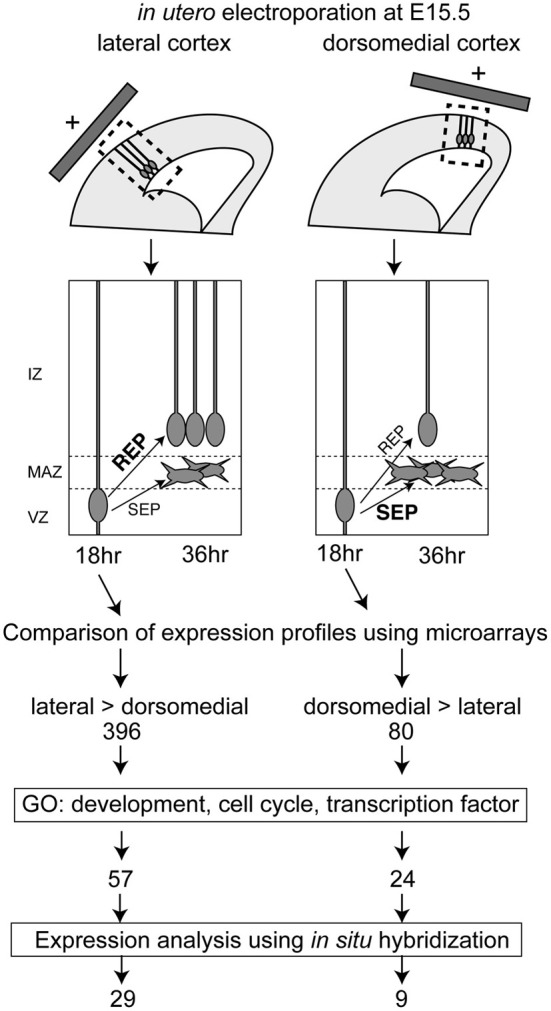
**Screening procedures for identifying genes involved in the regulation of the REP production rate and the number of genes selected in each step**. The REP production rate was high in the lateral VZ, compared with the dorsomedial region. Hence, the lateral and dorsomedial VZ cells labeled using GFP and *in utero* electroporation at E15.5 were collected 18 h after electroporation, when the cells undergo differentiation into REP or SEP. Microarray analyses identified 396 genes with a lateral-high and 80 genes with a medial-high expression pattern. The candidate genes were further selected based on gene ontology (GO) and were narrowed down to 62 candidate genes with lateral-high and 23 candidate genes with medial-high expression patterns. The expression patterns of these candidate genes were checked in the BGEM database or by performing *in situ* hybridization, and 29 genes with lateral-high and 9 genes with medial-high expression patterns were identified.

Because the ratio of the production of proliferative cells in the mouse IZ/SVZ (REP) to that of postmitotic cells (SEP) within the population exiting from the VZ is high in the lateral VZ and low in the dorsomedial VZ (Tabata et al., 2009), we compared the gene expression profiles between the lateral VZ and the dorsomedial VZ to screen for candidate genes regulating the REP/SEP production ratio in mouse cortex. We first introduced a GFP expression vector into the lateral or dorsomedial VZ at embryonic day (E) 15, and GFP-positive cells were collected by FACS 18 h later, by which time the fate of newly born cells in the VZ (whether they will belong to the REP or SEP) is thought to have been determined (Tabata et al., 2009) (Figure 1). We extracted RNA from the GFP-positive cells and performed transcriptome analyses using DNA microarrays. We selected probe sets with the detection call “present” (*P* < 0.05) in both lateral and dorsomedial VZ cells, since we had previously noticed that the REP production rate was low in the dorsomedial region but remained significant [the REP production rate among cells exiting the VZ was about 65% in the lateral VZ while that in the dorsomedial VZ was 40% when labeled at E15 (Tabata et al., 2009)]. We further selected probes for which the signals showed a high lateral, low dorsomedial pattern or the opposite pattern with an increased or decreased call (*P* < 0.05) using the MAS5 algorithm, followed by those with a threshold of a 2-fold change (1 log-fold change). As a result, we identified 396 and 80 probe sets with high lateral and high dorsomedial expression patterns, respectively. We then obtained the gene ontologies (GOs) for the probe sets and narrowed down the candidates to those with a GO related to development or the cell cycle. The reason why we chose a “cell cycle” GO is that since the SEP is postmitotic, whereas the REP is still proliferative, such a GO would be expected for genes involved in the decision to acquire an SEP fate or an REP fate among the VZ-derived cells. We also selected transcription factors to obtain potential key factors for the REP/SEP production ratio. In this step, we integrated multiple probe sets into one transcript. Using these procedures, we identified 62 and 23 genes with higher expressions in the lateral and dorsomedial VZ regions, respectively.

**Figure 2:**
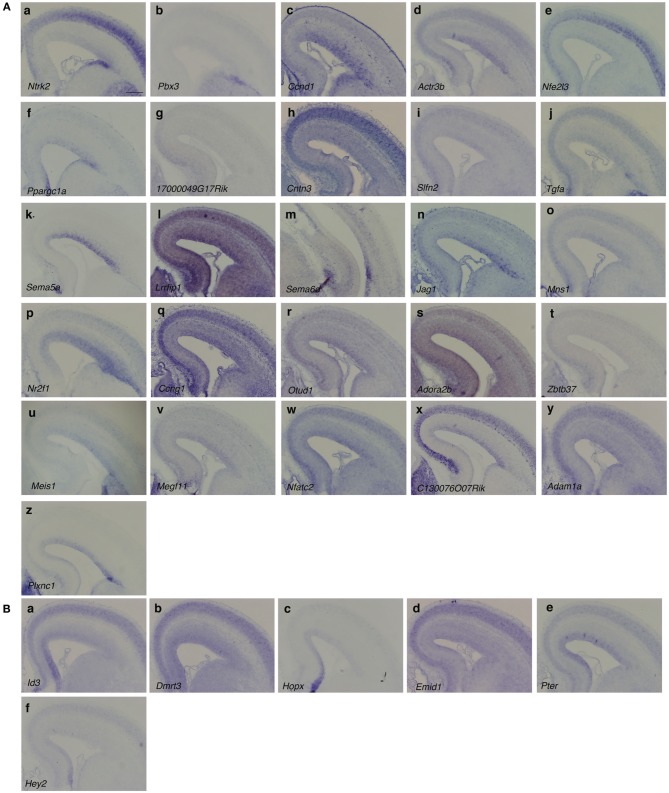
***In situ* hybridization of candidate genes**. The DIG-labeled RNA probes of the indicated genes were hybridized on the coronal section of E16 mouse brains. Genes with **(A)** lateral-high and **(B)** medial-high expression patterns are shown. Scale bar: 200 μm.

Next, we examined the expression profiles of these 85 candidate genes using an *in-situ* hybridization database, the Brain Gene Expression Map (BGEM; http://www.stjudebgem.org/web/mainPage/mainPage.php) (Magdaleno et al., 2006). Consequently, we found 5 lateral-high (*Fgfr2, Ntrk2, Pbx3, Pmp22, Ntng1*) and 5 dorsomedial-high (*Dmrt3, Id3, NeuroD4, Fzd9, Fbln7*) candidate genes in the BGEM database. *In-situ* hybridization experiments included in the BGEM database showed gradient expression patterns for E15.5 mouse forebrain that were consistent with our microarray results, except for those for the *Fbln7* gene (data not shown). For 2 lateral-high genes (*Ntrk2* and *Pbx3*) and 2 medial-high genes (*Dmrt3* and *Id3*), for which the expression patterns shown in the BGEM database appeared to be consistent with our microarray results, we further confirmed the expression gradient in the VZ using *in situ* hybridization with the RIKEN FANTOM cDNA clone set (Okazaki et al., 2002; Carninci et al., 2005) as templates for probe synthesis (Figures 2A,B).

For other candidates, we performed *in situ* hybridization to confirm the gradient expression in the VZ. Among the remaining 57 lateral-high and 18 medial-high genes, 49 and 16 genes were included in the cDNA clone set with an insert longer than 1 kilo-base pairs (kbp). We therefore performed *in situ* hybridization for these genes on sections of E16 mouse brains and found 24 lateral-high and 4 medial-high candidate genes. One lateral-high gene, *Sema6d*, showed a lateral-high to medial-low gradient only in the caudal region (Figure 2Am).

Overall, we identified 29 and 9 genes expressed in a lateral-high to medial-low or a medial-high to lateral-low gradient pattern in the VZ, respectively (Figure 1).

In this screening, we did not obtain well-known arealization factors (Borello and Pierani, 2010) as candidate genes. Among such genes, we were able to detect the expressions of Pax6, Emx2, Bmp2, and Wnt5a in both the lateral and dorsomedial VZ cells, but the differences in the expression levels between the lateral and dorsomedial VZs were not significant according to the MAS5.0 algorithm. As for Fgf8, Bmp4, and Wnt3a, the expressions of these genes were not detected in either the lateral or dorsomedial VZ cells. This observation might reflect the fact that we collected the VZ cells at a late stage of cortical plate development, at which time arealization had already been completed.

### Bioinformatics evolutionary analysis on the lateral-high and medial-high candidate genes

The 29 and 9 genes with lateral-high and medial-high expression patterns, respectively, can be considered as potential candidates responsible for the high REP/SEP production ratio in the mouse lateral cortex, compared with the dorsomedial cortex. These genes might include candidate molecules that were involved in the dramatic increase in the number of bRGs during primate cortical evolution. Thus, we then performed bioinformatics evolutionary analyses for these 38 genes. The evolutionary pressures on the candidate genes were estimated using evolutionary models, which are based on the synonymous and non-synonymous base substitution rates (dN/dS) of the coding DNA sequences.

**Figure 3:**
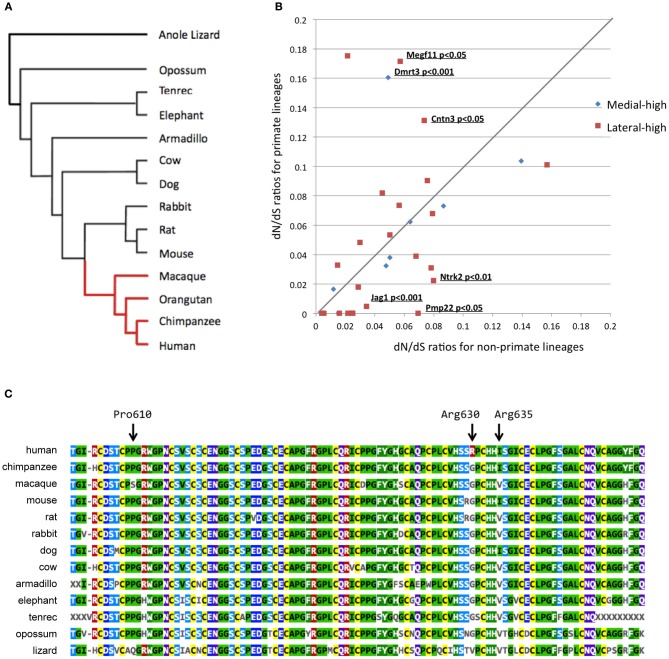
**Bioinformatics evolutionary analyses of 38 candidate genes. (A)** Phylogenetic tree used to perform the multiple alignments. The red and black lineages in the phylogenetic tree represent primate and non-primate lineages, respectively. **(B)** Two-dimensional plotting of the dN/dS ratios in primate and non-primate lineages. The X- and Y-axes represent dN/dS ratios for non-primate and primate lineages, respectively. The gene symbol and *P*-values are shown for differences between primate and non-primate lineages with a significance level of 0.05. **(C)** Alignment of the Megf11 protein sequences in 13 species. The orangutan Megf11 protein sequence was not included in this alignment because Ensembl did not contain any *Megf11* gene annotation for the orangutan genome, possibly because of the low quality of the genome. This alignment shows that Arg630 is a human-specific residue while the amino acid site is strongly conserved among mammalian species, except for humans.

To estimate the dN/dS ratios, we first investigated the one-to-one correspondence of candidate genes among 13 mammalian species and 1 reptile, the Anole lizard, as shown in Figure 3A. We excluded three candidate genes (*Slfn2*, 1700049G17Rik, and *Adam1a*) from subsequent evolutionary analyses because these genes were found to have undergone gene loss or gain events when mice and humans were compared.

To figure out evolutionary pressures of the remaining 35 candidate genes, we applied the “two-ratio” evolutionary model (see Materials and Methods) to the candidate genes. Figure 3B shows the dN/dS ratios of the 35 candidate genes in primate (y-axis) and non-primate (x-axis) lineages. The likelihood ratio test, which compares the “one-ratio” and “two-ratio” evolutionary models, suggested that the dN/dS ratios of three genes (*Jag1, Ntrk2*, and *Pmp22*) were significantly lower in primate lineages than in non-primate lineages (*P* < 0.05). These results indicated that the negative selection for *Jag1, Ntrk2*, and *Pmp22* in the primate lineages was stronger than that in the non-primate lineages, implying that the molecular functions of these three genes are likely to be more essential in primates than in non-primates. In contrast, the dN/dS ratios of three other genes (*Megf11, Dmrt3*, and *Cntn3*) were significantly higher in the primate lineages, compared with those in the non-primate lineages (*P* < 0.05).

Next, we performed additional detailed evolutionary analyses for *Megf11, Dmrt3*, and *Cntn3*. dN/dS ratios higher than 1.0 are indicative of adaptive molecular evolution (Hughes and Nei, 1988; Messier and Stewart, 1997; Zhang, 2003). Although the dN/dS ratios averaged over the whole amino acid residues of the *Megf11, Dmrt3* and *Cntn3* gene products were less than 1.0, we searched for amino acid residues with positive selection in primate lineages using branch-site evolutionary models (see Materials and Methods). For *Dmrt3* and *Cntn3*, our branch-site models yielded no residues under positive selection, i.e., the dN/dS ratios of all the residues were estimated to be smaller than 1.0. For *Megf11*, the branch-site models suggested that seven residues (Arg561, Pro568, Pro610, Arg630, Ile635, Ser802 and Met971) were under positive selection in primate lineages. Among the seven residues, Arg630 is human-specific (Figure 3C), Arg561 is chimpanzee-specific, Pro568 and Pro610 are macaque-specific, Ile635 is common among humans and chimpanzees, and Ser802 and Met971 are common among primates. Arg630 is harbored in the laminin-type EGF-like domain, which mediates cell adhesion, growth, migration and differentiation. These results suggested that *Megf11* underwent adaptive molecular evolution in primate lineages, implying that the molecular function of *Megf11* might be involved in primate-specific traits. Among the three genes with dN/dS ratios that differed significantly between primate and non-primate lineages, *Jag1* showed the most obvious primate-specific selection (*P* < 0.001).

## Discussion

In this study, we attempted to identify candidate molecules that were potentially involved in the expansion of the cerebral cortex during primate evolution. We previously reported that the ratio of REP production to SEP production in the mouse cortex was higher in the lateral VZ than in the dorsomedial VZ and that the mouse REP had characteristics similar to those of primate bRG cells. Specifically, both have a long ascending process and are distributed widely in the SVZ, overlapping with or superficially to the region with a high cell density located above the VZ (MAZ in mouse and ISVZ in primates), which is composed of randomly oriented cells. Moreover, mouse REP includes Sox2-positive and/or Pax6-positive bRG-equivalent cells as a minor population, in addition to BPs and glial progenitors. Based on these facts, we assumed that the molecules responsible for the high REP/SEP production ratio in the mouse lateral cortex, compared with the dorsomedial cortex, might include candidate genes that had contributed to the expansion of bRGs in the primate cortex during evolution. Therefore, we screened for genes that were expressed in a lateral-medial gradient pattern to identify candidate molecules involved in the determination of the REP/SEP production ratio in mouse cortex. We then further performed detailed evolutionary analyses of the resulting candidate genes. As a result, significant changes in evolutionary pressure between primate and non-primate lineages were found for six genes. These genes are known/suggested to be involved in cell proliferation, differentiation, and adhesion and might affect the REP/SEP production ratio in mice.

Of course, some of the candidate genes that were isolated might have simply reflected areal differences. Further analyses are needed to test the roles of candidate genes on the regulation of the REP/SEP production ratio. During cortical development, a neurogenic gradient exists from the lateral to dorsomedial region. Hence, the difference in the REP/SEP production ratio between the lateral cortex and the dorsomedial cortex might reflect differences in the developmental stage. However, we previously showed that the REP production rates of the VZ cells labeled at E15 and E16 were not dramatically different from each other (65 and 68%, respectively) (Tabata et al., 2009). Therefore, we assumed that the higher REP/SEP production ratio in the lateral cortex, compared with that in the dorsomedial cortex, was unlikely to be simply due to the differences in the neurogenic stages, but to have been largely caused by the need to satisfy the regional demand for neuron production.

The significance of Notch signaling in the proliferation of bRG cells has been previously reported (Hansen et al., 2010). Considering that the mouse REP is a proliferative population, while the SEP is a postmitotic population, Jag1, a conventional ligand of Notch receptors, is an important candidate that might have evolved to increase the REP/SEP production ratio in primates. Megf11 is a member of the MEGF family, which includes Megf10 and Jedi-1 in mammals and CED-1 in *C. elegans*. Although MEGF family proteins were initially shown to mediate engulfment as a binding protein for cell corpses (Zhou et al., 2001; Wu et al., 2009), a later study demonstrated that MEGF family proteins also act as a Notch signaling modifier (Krivtsov et al., 2007). MEGF family proteins have a weak homology to conventional Notch ligands, such as Jag1, including an N-terminal DSL-like domain and multiple EGF-like repeats. Krivtsov et al. demonstrated that MEGF family proteins inhibit Notch signaling, probably by binding directly to Notch receptors. The positive selection residues of the *Megf11* gene product were mainly harbored in the 11–14th EGF-like repeat. Thus, it would be interesting if the affinity with Notch receptors is changed in primates. Further analyses are needed to clarify the meaning of the primate-specific positive selections on the *Megf11* gene.

The role of the genes identified here, especially on the potential regulation of the REP/SEP production ratio, in mice and whether evolutionary changes in these genes were involved in the expansion of the OSVZ in primates should be clarified in future studies.

### Conflict of interest statement

The authors declare that the research was conducted in the absence of any commercial or financial relationships that could be construed as a potential conflict of interest.
